# Public Knowledge and Perceptions of Fentanyl Test Strips: A National Cross-Sectional Survey Informed by the Health Belief Model

**DOI:** 10.3390/healthcare14070833

**Published:** 2026-03-24

**Authors:** Lindsey Hohmann, Madelynn Aeilts, Caitlyn Johnson, Gabriela Rajic, Annarose Sorvillo, Brandy Davis, Anne Taylor

**Affiliations:** 1 Harrison College of Pharmacy, Auburn University, Auburn, AL 36849, USA; brd0001@auburn.edu (B.D.);; 2 Drake University College of Pharmacy and Health Sciences, Des Moines, IA 50311, USA; 3 School of Pharmacy, South University, Savannah, GA 31406, USA; 4 Midwestern University at Glendale College of Pharmacy, Glendale, AZ 85308, USA; 5 Fairleigh Dickinson University School of Pharmacy and Health Sciences, Florham Park, NJ 07932, USA; ams23@student.fdu.edu

**Keywords:** fentanyl, survey, harm reduction, perceptions, intention, Health Belief Model

## Abstract

**Background/Objectives**: Fentanyl test strips (FTS) are a harm reduction tool used to detect fentanyl in illicit substances. However, little is known regarding Americans’ beliefs regarding FTS. Therefore, the purpose of this study was to assess the U.S. general public’s FTS knowledge and perceptions. **Methods**: This study utilized a cross-sectional design. Adults ≥18 residing in the U.S. were recruited to participate in an anonymous online survey via Amazon Mechanical Turk (MTurk). Participants received $5 upon survey completion. The survey instrument was informed by the Health Belief Model, and primary outcome measures included: (1) FTS knowledge (13-items); (2) perceived susceptibility to fentanyl exposure (8-items); (3) perceived severity of fentanyl exposure (10-items); (4) perceived FTS benefits (9-items); (5) perceived barriers to FTS access (13-items); (6) comfort using and accessing FTS (15-items); (7) confidence using and accessing FTS (11-items); and (8) FTS utilization intentions (6-items). Outcomes were measured via 5-point Likert-type scales (1 = strongly disagree, 5 = strongly agree). Data were analyzed using descriptive statistics and Mann–Whitney U tests to compare differences in scale scores across participant sociodemographics. Predictors of FTS utilization intentions were assessed via multiple linear regression, controlling for participant age, race, sex, geographic setting (rural/urban), and recreational drug use history (yes/no) (α = 0.05). **Results**: Of *n* = 206 respondents, the majority were male (55.8%) and White (83.0%) with a mean age of 46.4. Approximately 81% resided in urban areas and 58.5% reported a history of recreational drug use. Participants who identified as Black, Asian, Indigenous, Pacific Islander, or Multiracial reported significantly higher mean (SD) perceived susceptibility compared to White participants (2.06 [0.54] vs. 1.91 [0.58]; *p* = 0.034). Participants residing in urban areas reported significantly higher comfort using and accessing FTS (3.61 [0.86]) than those in rural areas (3.29 [0.92]; *p* = 0.048), and younger individuals (≤44.5 years) were more confident in their ability to access FTS (3.75 [0.73]) compared to their older counterparts (3.60 [0.64]; *p* = 0.048). Perceived susceptibility (β = 0.442; *p* < 0.001), benefits (β = 0.250; *p* = 0.020), and comfort (β = 0.453; *p* < 0.001) were positive predictors of FTS utilization intention (R^2^ = 0.417). **Conclusions**: Perceptions regarding FTS varied across race, geographic setting, and age. Perceived susceptibility, perceived benefits, and comfort positively predicted the U.S. general public’s FTS utilization intentions. Future interventions may leverage these influential factors to enhance FTS uptake.

## 1. Introduction

Fentanyl, a synthetic opioid that is up to 100 times more potent than morphine, has become a leading contributor to the escalating overdose crisis in the United States [1,2]. In 2022 alone, over 70,000 overdose deaths were attributed to synthetic opioids, primarily fentanyl, marking a sharp increase in drug-related mortality over the past decade [1]. The widespread and often unintentional presence of fentanyl in the illicit drug supply poses a unique challenge for overdose prevention, as individuals may unknowingly consume fentanyl-contaminated substances, increasing their risk of fatal overdose [3]. To mitigate this risk, fentanyl test strips (FTS) have emerged as a low-cost, accessible harm reduction tool that allows people who use drugs to test substances for the presence of fentanyl before use [4].

Evidence from studies involving people who use drugs (PWUD) suggests that FTS are highly acceptable and perceived as easy to use, especially when accompanied by brief educational interventions [5,6,7]. PWUD who utilize FTS report greater awareness of overdose risks, and in many cases, self-reported changes in drug use behaviors based on test results (e.g., carrying naloxone, discarding drugs, not using alone) [8,9,10]. Furthermore, interest in learning how to use FTS is often driven by personal experiences or concern for the well-being of friends and family, indicating that motivation for harm reduction extends beyond individual drug use [7,9]. These findings point to the potential value of expanding access to FTS as a public health strategy, especially when integrated with education and counseling services.

Community-based and pharmacy-centered studies also highlight the role healthcare professionals can play in FTS distribution [11,12]. For example, pharmacists in one statewide survey expressed willingness to engage in conversations about FTS use, provide counseling, and refer individuals to local harm reduction organizations, provided they receive appropriate training and institutional support [12]. These findings illustrate the growing interest in mainstreaming FTS availability and point toward a broader application of harm reduction principles in traditional healthcare settings.

Despite this growing body of evidence, existing research has primarily focused on high-risk or substance-using populations, such as PWUD, individuals receiving addiction treatment services, or participants recruited from syringe service programs [5,6,7,8,9,10]. As such, little is known about the U.S. general public’s baseline knowledge, beliefs, and perceptions of fentanyl test strips. This represents a critical gap in the literature, particularly as efforts to combat the opioid crisis increasingly call for widespread public education and community-based prevention strategies [13].

Therefore, the purpose of this study was to assess the general U.S. public’s knowledge, perceptions, and intentions regarding fentanyl test strips. By sampling the general U.S. public rather than a PWUD-focused setting, this study complements prior research [5,6,7,8,9,10] and informs population-wide communication. Specifically, by identifying key gaps in understanding and measuring attitudes toward FTS among a broader audience, this research seeks to inform the development of future public health policies and interventions that are inclusive, scalable, and rooted in community education. Understanding public perception is essential for shaping effective messaging, reducing stigma, and expanding access to life-saving tools aimed at reversing the trajectory of the opioid epidemic.

## 2. Materials and Methods

### 2.1. Study Design and Participant Recruitment

This study utilized a cross-sectional design. Adults ≥18 residing in the U.S. were recruited to participate in an anonymous online survey via Amazon Mechanical Turk (MTurk) in November–December 2024. MTurk is an online crowdsourcing platform that enables reach to both national and international audiences [14]. Previous studies have demonstrated that MTurk users are similar to the demographics of the U.S. as a whole in terms of age, sex, and race [15]. Given the nationwide reach and representation of a breadth of demographic groups, MTurk was utilized in the current study to enable efficient recruitment of the U.S. general public. In order to ensure receipt of high-quality responses, individuals without the MTurk “Masters” designation, which indicates a history of reliable work, were excluded from participation [16]. Participants received $5 upon survey completion.

The minimum sample size for this study was determined via an a priori power calculation using G*Power software version 3.1.9.7 (Heinrich-Heine-Universität, Düsseldorf, Germany) [17,18]. Assuming a medium effect size (f^2^ = 0.15) [19] and an alpha of 0.05, a minimum sample size of 127 was deemed sufficient to determine predictors of FTS utilization intention (the primary outcome measure of interest) via multiple linear regression with 80% power. The final sample size exceeded the minimum requirement (see Section 3).

### 2.2. Data Collection and Measures

The survey was distributed to eligible participants electronically via MTurk. The survey instrument was guided by the Health Belief Model, which posits that perceived susceptibility, severity, benefits, and barriers surrounding a health condition and preventive/therapeutic services influence an individual’s health-related behavior [20]. Survey measures were pre-tested for face and content validity among *n* = 3 members of the corresponding authors’ department. Primary outcome measures included: (1) objective FTS knowledge (13-items) and subjective (self-rated) FTS knowledge (6-items); (2) perceived susceptibility to fentanyl exposure and use risks (8-items); (3) perceived severity of fentanyl exposure and use risks (10-items); (4) perceived FTS benefits (9-items); (5) perceived barriers to FTS use and access (13-items); (6) comfort using and accessing FTS (15-items); (7) confidence (self-efficacy) in abilities to use and access FTS (11-items); and (8) FTS utilization intentions (6-items). Outcomes were measured via multiple-choice (objective knowledge) and 5-point Likert-type scale questions (1 = strongly disagree, 5 = strongly agree). Objective knowledge questions were informed by previously published literature from the United States Drug Enforcement Administration (DEA) [21,22], Centers for Disease Control and Prevention (CDC) [4], Substance Abuse and Mental Health Services Administration (SAMHSA) [23], health departments [24,25], and others [26,27,28,29,30]. Likert-type scale items were informed by work published by Goldman [9], Peiper [8], Tilford [31], Reed [1,32], Bonar [33], and Barrolle [7] and colleagues.

Additionally, geographic setting (rural/urban), awareness of FTS prior to the current survey (yes vs. no), FTS usage history (yes vs. no), and recreational drug use history (yes/no) were assessed. Rural versus urban location was assessed via zip codes using the Rural Urban Commuting Area (RUCA) codes, with RUCA codes 1–3 corresponding to urban and codes 4–10 corresponding to rural locations [34]. Recreational drug use history was operationalized as any lifetime non-medical drug use and was captured via a single dichotomous (yes/no) multiple-choice question (“*Have you ever used recreational drugs, excluding alcohol, tobacco, or caffeine, for purposes other than those required for medical reasons?*”) adapted from the Drug Abuse Screening Test (DAST-10) [35,36]. The full survey instrument is available in Supplemental File S1. All study procedures received ethical approval via the primary author’s Institutional Review Board (Protocol #STUDY00000088), and all respondents provided consent to participate.

### 2.3. Data Analysis

Data were analyzed using descriptive statistics (means, standard deviations, frequencies, and percentages). An overall objective knowledge score was calculated based on the total number of multiple-choice questions answered correctly (percent correct), while Likert-type scale items were summed and averaged to create total mean scale scores for subjective knowledge, perceived susceptibility, severity, benefits, barriers, comfort, and confidence. Likert-type scale items were reverse-coded as necessary prior to calculation of mean scale scores, and internal consistency of scales was assessed via KR-20 (objective knowledge) and Cronbach’s alpha. Missing item-level responses were handled by excluding those items from analysis, and respondents who did not complete at least 80% of survey items were removed from the dataset. Due to limited numbers of participants in several sociodemographic subgroups, including race, sociodemographic variables were collapsed into binary categories to preserve statistical power. Mann–Whitney U tests (α = 0.05) were used to compare differences in mean scale scores across dichotomized participant sociodemographics, including age (≤respondents’ median age vs. >the median age), race (Black, Asian, Indigenous, Pacific Islander, Multiracial, or Other vs. White), sex (male vs. female), geographic setting (rural vs. urban), awareness of FTS prior to the current survey (yes vs. no), FTS usage history (yes vs. no), and recreational drug use history (yes vs. no). Non-parametric analyses (Mann–Whitney U tests) were utilized given that the scale-level data did not meet normality assumptions (Kolmogorov–Smirnov *p* < 0.05).

Furthermore, predictors of FTS utilization intentions were assessed via multiple linear regression. Regression models included objective knowledge, susceptibility, severity, benefits, barriers, comfort, and confidence mean scale scores as predictors (unadjusted model), controlling for participant age, race, sex, geographic setting, and recreational drug use history (adjusted model). Multiple linear regression was utilized given the approximate multivariate normality and collective linearity of the distribution of standardized residuals and predicted values, as well as the lack of autocorrelation (Durbin–Watson = 1.94), multicollinearity (VIF < 5), influential outliers (Cook’s Distance < 1), or heteroskedasticity (modified Breusch–Pagan test *p* = 0.255).

## 3. Results

### 3.1. Participant Characteristics

Of 208 respondents, a total of 206 participants completed the survey and were retained in the analytic dataset. The majority of respondents were White (83%), male (55.8%), and employed full-time (69.9%) (Table 1). The mean age was 46.39 years (median: 44.5), with 40.8% having a bachelor’s degree and 13.6% without health insurance. Respondents were located across 42 U.S. states in primarily urban locations (81.5%), with the highest representation from California (12.6%), Florida (9.2%), and Pennsylvania (7.3%). Study participants were not statistically significantly different than the general U.S. population in terms of race and sex (75.3% White and 50.5% female nationally) [37]. However, participants were significantly older than the average American (national mean age of 38.9 years) (t = 8.898, *p* < 0.001) [38]. Furthermore, 33.3% of respondents were aware of FTS prior to the current survey, 1.5% had used FTS previously, and 58.3% reported a lifetime history of recreational drug use.

### 3.2. Knowledge

Overall, respondents correctly answered a mean (SD) of 52.73% (25.69) of objective knowledge questions (Table 2a) (KR-20 = 0.826). Similarly, respondents’ self-reported subjective knowledge was low, with a mean (SD) scale score of 2.47 (8.0) (Cronbach’s alpha = 0.755) (Table 2b). In particular, 67.9% of individuals agreed and strongly agreed that they needed more knowledge about how to use FTS.

Analysis of demographic subgroups (Table 3) revealed significantly higher mean (SD) objective (65.04 [47.32] vs. 47.32 [26.64]; *p* < 0.001) and subjective (2.66 [0.83] vs. 2.38 [0.77]; *p* = 0.012) knowledge scores among those aware versus not aware of FTS. Similarly, participants with a history of FTS usage had higher objective (84.62 [13.32] vs. 52.40 [25.57]; *p* = 0.025) and subjective (4.07 [1.00] vs. 2.44 [0.77]; *p* = 0.012) knowledge compared to those with no FTS usage history. No significant differences in objective or subjective knowledge scores were found based on recreational drug use history, geographic location, sex, race, or age.

### 3.3. Perceived Susceptibility

Perceived susceptibility to fentanyl exposure and use risks was low among participants (Table 3), with a mean (SD) overall score of 1.93 (0.57) (Cronbach’s alpha = 0.676). Responses to individual items within the scale further contextualize this finding (Supplemental File S2, Table S1). The majority of participants strongly disagreed with the statement, “I will likely overdose on fentanyl in my lifetime” (79.1%), and 80.5% strongly disagreed with having knowingly or unknowingly used fentanyl. A substantial portion also reported no familiarity with fentanyl access, with 71.6% strongly disagreeing with knowing where to purchase fentanyl. Regarding community-level risk, 52.2% of respondents disagreed or strongly disagreed that fentanyl overdoses are common in their communities, though 18.4% agreed or strongly agreed with this statement. Nearly half of participants (47.2%) agreed or strongly agreed with the statement “I do not have to worry about overdosing on fentanyl.” Only 8.1% expressed any level of agreement with the belief that they would not overdose even if they used fentanyl.

Subgroup analyses revealed several significant differences in perceived susceptibility (Table 3). Participants who identified as Black, Asian, Indigenous, Pacific Islander, or Multiracial reported significantly higher mean (SD) perceived susceptibility compared to White participants (2.06 [0.54] vs. 1.91 [0.58]; *p* = 0.034). Similarly, individuals who were aware of FTS reported higher susceptibility than those who were not (2.09 [0.60] vs. 1.84 [0.54]; *p* = 0.003). Those with a history of FTS use reported the highest perceived susceptibility (3.47 [0.52]), which was significantly greater than that of those without such a history (1.91 [0.54]; *p* = 0.004). Additionally, individuals with a history of recreational drug use perceived themselves to be more susceptible (2.07 [0.63]) compared to those without drug use history (1.75 [0.43]; *p* < 0.001). No statistically significant differences in perceived susceptibility existed based on rural versus urban residence, gender, or age group.

### 3.4. Perceived Severity

Overall, perceived severity of fentanyl exposure and use risks was high (Table 3), with a mean (SD) score of 4.63 (0.38) (Cronbach’s alpha = 0.824). Item-level responses revealed widespread recognition of fentanyl’s potential to cause serious harm (Supplemental File S2, Table S2). A large proportion of participants strongly agreed that fentanyl can cause a fatal overdose (82.6%), non-fatal overdose (39.8%), and serious harm (83.0%). Furthermore, nearly all respondents agreed or strongly agreed that using fentanyl could lead to substance use problems (100%), with 78.9% strongly endorsing this belief.

Participants also expressed strong agreement with statements linking fentanyl use to a range of negative life outcomes. Specifically, 75.6% strongly agreed that fentanyl use could impair the ability to maintain employment, 75.2% that it could lead to strained relationships with friends or family, 75.0% that it could cause legal problems, and 76.6% that it could result in financial difficulties. Additionally, 68.2% strongly agreed that fentanyl is highly addictive. There was greater variation in responses regarding the belief that fentanyl use can cause skin infections, with 39.1% indicating a neutral response and only 31.8% strongly agreeing. No statistically significant differences in perceived severity existed between subgroups (Table 3).

### 3.5. Perceived Benefits

Participants expressed a high level of agreement with statements related to the positive impact of FTS (Table 3), with a mean (SD) overall perceived benefits score of 4.15 (0.84) (Cronbach’s alpha = 0.899). Several specific statements related to public health and safety received particularly strong support (Supplemental File S2, Table S3). The highest level of agreement reported was that “FTS saves lives” (58.1% strongly agreed). Additionally, 57.1% of participants strongly agreed that FTS improves safety for people who use drugs, and 57.3% strongly agreed that FTS improves the safety of law enforcement officers. In contrast, perceived benefits related to behavior change and recovery received lower levels of agreement; 21.8% of participants strongly agreed that FTS supports recovery from drug addiction, and 27.8% strongly agreed that FTS can lead to positive changes in a person’s drug use. Mean perceived benefits scores did not differ significantly across subgroups (Table 3).

### 3.6. Perceived Barriers

Overall, mean (SD) perceived barriers to FTS use and access were low (2.53 [1.14]) (Cronbach’s alpha = 0.909) (Table 3). However, item-level responses revealed a range of perspectives (Table 4). For example, several items reflected a general disagreement with logistical or practical barriers. A majority of respondents disagreed or strongly disagreed with the statement that “FTS is too expensive” (80.5%). Similarly, the idea that “using FTS would take too much time” was dismissed by many (81.4% disagreed or strongly disagreed). Other commonly rejected barriers included the notion that “there is a lack of space where FTS could be conveniently used” (71.1% disagreed or strongly disagreed) and that “FTS are too difficult to use” (74.4% disagreed or strongly disagreed).

In contrast, other items reflected a notable level of concern and agreement, indicating that some barriers remain highly relevant. The most widely endorsed barrier was a lack of knowledge about how to use FTS (64.5% agreed or strongly agreed). In addition, concerns about the reliability of FTS purchased online were prominent (53.3% agreed or strongly agreed). Similarly, availability in physical retail locations was also a concern, with 50.9% agreeing or strongly agreeing that FTS is not readily available in stores or pharmacies. Mean perceived barriers scores did not differ significantly across subgroups (Table 3).

### 3.7. Comfort

Mean (SD) comfort with using and accessing FTS was positive overall (3.55 [0.87]) (Cronbach’s alpha = 0.935) (Table 3), but item-level analysis revealed areas of discomfort (Supplemental File S2, Table S4). In particular, while 68.9% of participants agreed or strongly agreed that they would trust the results of FTS, 41.4% disagreed or strongly disagreed that they would not feel embarrassed to ask about FTS. Furthermore, although most individuals agreed or strongly agreed that they felt comfortable asking their doctor (56.2%) and local pharmacist (50.0%) about FTS, comfort with purchasing FTS was highest from online retailers (71.8%) when compared to physician offices (63.9%) and local pharmacies (55.0%).

Additionally, participants residing in urban areas reported significantly higher mean (SD) comfort using and accessing FTS (3.61 [0.86]) than those in rural areas (3.29 [0.92]; *p* = 0.048) (Table 3). There were no statistically significant differences in comfort across other subgroups.

### 3.8. Confidence

Overall, participants’ mean (SD) confidence in their ability to use and access FTS was positive (3.67 [0.69]) (Cronbach’s alpha = 0.861) (Table 3). Interestingly, although only 27.8% agreed or strongly agreed that they knew where to purchase FTS, 67.3% felt confident in their ability to find locations where FTS is sold (Supplemental File S2, Table S5). Furthermore, while 32.5% disagreed or strongly disagreed that they felt confident in their ability to use FTS, 83.8% were confident in their ability to find more information about FTS. Of note, 73.4% agreed or strongly agreed that they were confident in their ability to decipher FTS results, but fewer (53.2%) knew how to proceed after receiving results from FTS.

There were significant differences in confidence accessing FTS across subgroups (Table 3). Participants who had previously heard of FTS reported significantly higher mean (SD) confidence (3.84 [0.70]) than those who had not (3.60 [0.68]; *p* < 0.010). Further, younger individuals (≤44.5 years) were more confident in their ability to access FTS (3.75 [0.73]) compared to their older counterparts (3.60 [0.64]; *p* = 0.048). There were no statistically significant differences in confidence across other subgroups.

### 3.9. Intentions

Collective FTS utilization intentions were low (mean [SD] score: 2.78 [0.94]) (Cronbach’s alpha = 0.874) (Table 3). Approximately 4% of respondents agreed or strongly agreed that they intended to obtain FTS in the next three months; 69.18% would recommend FTS to others at risk; and 47% were receptive to utilizing FTS (Supplemental File S2, Table S6). Of note, significant differences were found based on respondents’ history of recreational drug use (*p* = 0.041), with those having a history of recreational drug use stating a higher mean (SD) FTS utilization intention (2.90 [0.91]) compared to those without such history (2.61 [0.97]). In contrast, no significant differences in intentions existed across other subgroups.

**Figure 1:**
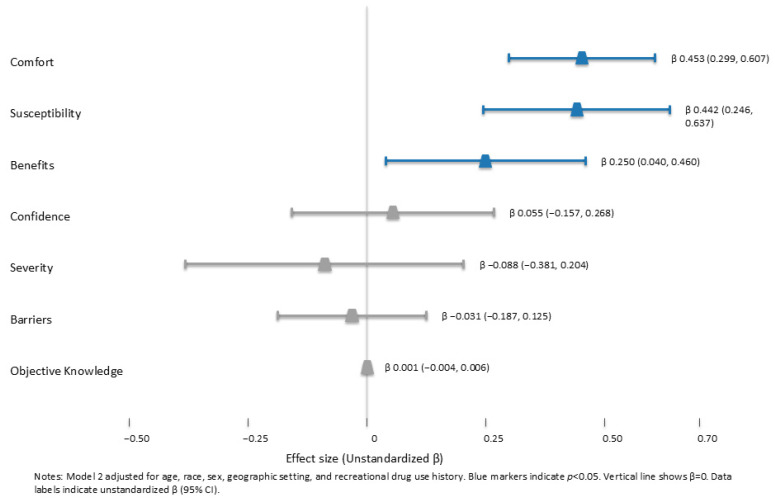
Predictors of FTS utilization intention (Model 2). Forest plot shows unstandardized β (points) and 95% CIs (horizontal bars) for objective knowledge, perceived susceptibility, perceived severity, perceived benefits, perceived barriers, comfort, and confidence after adjustment for age, race, sex, geographic setting, and recreational drug use history. Susceptibility, benefits, and comfort were statistically significant (*p* < 0.05).

Furthermore, influential factors associated with FTS utilization intentions were assessed via multiple linear regression (Table 5). In adjusted analysis (Model 2, Figure 1), three scales had a statistically significant influence on intention. Specifically, perceived susceptibility was a positive predictor of intention (β = 0.442, 95% CI = 0.246, 0.637; *p* < 0.001), such that those who perceived themselves as more susceptible to fentanyl exposure and use risks were more willing to use FTS. Perceived FTS benefits (β = 0.250, 95% CI = 0.040, 0.460; *p* = 0.020) and comfort in using and accessing FTS (β = 0.453, 95% CI = 0.299, 0.617; *p* < 0.001) were also positive predictors of intention, such that those who perceived greater benefits of FTS and were more comfortable using and accessing FTS were more willing to use FTS. Controlling for covariates did not alter model outcomes compared to unadjusted analysis (Model 1).

## 4. Discussion

This study revealed substantial educational gaps regarding FTS among participants. Approximately half of the objective knowledge questions (52.73%) were answered correctly, and self-reported (subjective) knowledge was low. Prior research has similarly documented low FTS knowledge. For example, Mistler et al. [39] found that 53% of individuals receiving medication for opioid use disorder reported no prior FTS knowledge, and Reed et al. found participants in a qualitative interview study making erroneous conclusions regarding FTS results, highlighting the need for basic FTS training [1]. Additionally, the current survey study found significant differences in both objective and subjective knowledge scores based on prior FTS awareness and FTS usage history. This indicates that prior FTS exposure plays a role in FTS understanding. However, no significant differences in knowledge scores were found based on recreational drug use history, suggesting that drug use does not inherently correlate with FTS understanding. This finding is particularly important as previous research has alluded to changes in drug use behavior upon identifying fentanyl present in illicit drugs [9]. These behavioral shifts highlight the potential impact of FTS when individuals are empowered with knowledge of available harm reduction tools. Taken together, this points toward the critical need for educational campaigns to enhance FTS utilization in the United States. In particular, given the moderate objective knowledge but low subjective knowledge reported in the current study, future educational interventions should prioritize practical, step-by-step instruction and interpretation guidance, ideally delivered in trusted retail/clinical settings (e.g., pharmacies) to bridge the gap from theoretical understanding to applied skills.

Furthermore, the current study revealed a notably low perceived susceptibility to fentanyl exposure and overdose among participants, despite widespread acknowledgment of the drug’s severity. A similar pattern emerged in a study by Moallef et al. in Vancouver, where 93.9% of PWUD possessed fentanyl-risk knowledge, yet 72.5% (comprising 35.0% who perceived no risk and 37.5% who perceived low risk) believed their own likelihood of overdosing was negligible [40]. These findings reinforce the idea that knowledge alone does not necessarily translate into perceived personal vulnerability, highlighting a persistent gap in risk-communication and overdose-prevention strategies.

Additionally, subgroup analyses indicated that Black, Asian, Indigenous, Pacific Islander, and Multiracial participants, individuals aware of fentanyl test strips (FTS), and those with a history of recreational drug use reported higher perceived susceptibility. These findings align with research suggesting that harm reduction tools like FTS can heighten individuals’ awareness of overdose risks and influence safer drug use practices. For example, a study by Peiper et al. [8] found that people who inject drugs who received a positive FTS result had significantly higher odds of changing their drug use behavior, such as using smaller amounts or administering drugs more cautiously, and reported feeling better able to protect themselves from overdose. This supports that FTS use not only facilitates behavioral change but may also contribute to heightened perceived susceptibility among those exposed to overdose risk.

Despite overall low perceived susceptibility, participants in the current study recognized the severe consequences of fentanyl use, with high agreement on its potential to cause fatal and non-fatal overdoses, addiction, and socio-economic problems. This acknowledgment of severity, coupled with low perceived personal risk, may be explained by the Protection Motivation Theory [41], which assumes that individuals assess both the severity of a threat and their vulnerability to it when deciding on protective behaviors. This suggests that while the perceived severity of fentanyl exposure is high, the perceived vulnerability remains low, potentially hindering the adoption of protective behaviors among the U.S. general public.

Additionally, the findings from this survey highlight a strong overall recognition of the benefits of FTS, alongside several persistent barriers that may limit their widespread use. Participants generally expressed high levels of agreement with statements related to the safety and harm reduction value of FTS. The most strongly supported benefits included the belief that FTS saves lives, improves safety for people who use drugs and enhances safety for law enforcement officers. These results align with growing public health messaging around FTS as a practical and effective tool in reducing opioid overdose risk [4]. However, when asked about more long-term or recovery-oriented benefits of FTS, such as supporting addiction recovery or encouraging positive changes in drug use, participants expressed comparatively lower levels of strong agreement. This may reflect uncertainty about whether FTS, while helpful in preventing overdose, are sufficient on their own to motivate behavior change or support sustained recovery. These nuanced perceptions suggest that while FTS are widely accepted as a life-saving intervention, public understanding of their broader role in care is still developing.

In contrast, responses regarding perceived barriers to FTS use revealed a mixed picture. Many participants strongly disagreed with practical barriers, such as costs, difficulty of use, time constraints or lack of appropriate space. On the other hand, some issues emerged as more prominent concerns. Notably, a majority of participants reported that lack of knowledge about how to use FTS was a significant barrier, and many expressed concerns about the reliability of products purchased online and limited availability in local stores or pharmacies. This aligns with prior research that found lack of knowledge on how to interpret FTS results and limited distribution locations to be major barriers [1]. Interestingly, Reed and colleagues found lack of an appropriate space to use FTS as a major barrier among PWUD in Philadelphia, but this did not emerge as a significant barrier in the current study [1]. Further research should investigate regional, sociodemographic (e.g., housing situation), or other contextual factors (e.g., type of recreational drug use) contributing to this barrier. Overall, the current study’s findings suggest that while physical and financial access may not be widely perceived as problematic, informational and distribution-related barriers persist. This points to an opportunity for public health efforts to focus on education, outreach and improving availability in trusted retail or clinical settings such as pharmacies.

Furthermore, findings from this study indicate that comfort and confidence in using FTS vary based on location, prior knowledge of FTS, and age. Specifically, urban residents, those previously aware of FTS, and younger individuals (≤44.5) reported significantly greater confidence in their use, underscoring the need for targeted education efforts in rural areas, middle-aged or older adults, and among populations with lower education levels or barriers to educational access. For pharmacists, these results highlight their critical role in harm reduction by providing patient education, increasing FTS accessibility in rural regions that may lack other healthcare providers, and addressing health literacy barriers [42]. Further, pharmacists can support public health efforts by stocking FTS, offering counseling on proper use, and advocating for policy changes that expand access to harm reduction tools [12]. Expanding pharmacist-driven harm reduction initiatives could bridge knowledge gaps, enhance community engagement, and thereby improve patient comfort and confidence in using and accessing FTS by normalizing FTS provision.

Interestingly, this study reveals a gap between endorsement and personal uptake of FTS. Nearly 70% of participants reported willingness to recommend FTS to others; however, only 4% reported intentions to obtain FTS in the next three months even though over half of participants reported a history of recreationally using drugs. These findings mirror behavior translation challenges seen in public health campaigns [43], emphasizing the critical need for future interventions to address this gap. Given that advocates with harm reduction experience have emphasized that messaging is more effective when it reflects community-specific values and frames harm reduction as an integral component to address drug use and its complications [44], targeted messaging campaigns may represent a potential avenue for future investigation.

Additionally, significant differences in FTS utilization intention were found based on respondents’ history of recreational drug use. This finding aligns with Bandara et al. [45], who observed significantly higher FTS use among individuals with a recent overdose history compared to those without (36.8% vs. 23.5%; *p* <0.001). Conversely, intent did not differ based on demographic factors such as age, sex, race, and geographic location. This may reflect the influence of access-related barriers, as this study, and others [45], find that lack of harm reduction awareness continues to hinder broader adoption.

Importantly, this study identified several factors that significantly influence FTS utilization intentions. Individuals who believed they were more likely to encounter fentanyl contamination and harm were more inclined to utilize FTS. This finding aligns with constructs from the Health Belief Model, which suggests that individuals who perceive health threats are more likely to adopt preventive health behaviors [46]. Other significant positive predictors of FTS utilization included beliefs in FTS benefits and comfort in using FTS, suggesting that both cognitive and practical confidence play a role in harm-reduction behavior.

Overall, effectively increasing FTS utilization may require more than general knowledge and awareness campaigns. Community pharmacists are uniquely positioned to support community-based strategies that address behavioral contexts and practical barriers. Pharmacists can play a vital role by offering confidential guidance, demonstrating FTS use, and reinforcing the value of harm reduction services [47,48]. In doing so, pharmacists can empower individuals to act on their intentions and promote public health strategies in reducing overdose deaths.

### Limitations

This study has several limitations to consider. First, the cross-sectional nature of the survey limited the causal conclusions that can be drawn. Future studies may wish to assess changes in FTS knowledge and perceptions among the U.S. general public over time. Further, social desirability and selection biases must be taken into account; however, the survey was designed to be anonymous to mitigate these concerns. Likewise, MTurk-based recruitment utilizing the “Masters” qualification may introduce a bias towards recruitment of professional survey-takers, potentially contributing to the older age and majority of urban residents among participants. This older age distribution and under-representation of rural residents observed in the current MTurk sample may attenuate generalizability for community-level risk perception and access-confidence outcomes. Future work should oversample younger adults and rural communities and consider community-partnered recruitment beyond crowdsourcing to balance these skews. Additionally, collapsing race to a binary variable in the current survey improved statistical power but reduced nuance; future studies should purposively recruit to analyze more granular racial/ethnic strata.

In addition, recreational drug use was operationalized in the current study as any lifetime non-medical drug use rather than current use, which should be considered when interpreting the relatively high endorsement of recreational drug use history by survey respondents. Further, the current study sampled the U.S. general public and not a PWUD-specific cohort, and direct comparisons to studies of PWUD should therefore be interpreted cautiously.

## 5. Conclusions

Perceptions regarding FTS varied across race, geographic setting, and age, as well as prior FTS awareness and recreational drug-use history. Perceived susceptibility, perceived benefits, and comfort positively predicted the U.S. general public’s FTS utilization intentions. Future interventions may leverage these influential factors to enhance FTS uptake.

## Figures and Tables

**Table 1:** Respondent characteristics (*n* = 206).

Demographics	*n* (%) ^a^
Gender Male Female	115 (55.8%) 89 (43.2%)
Race White Black or African American Asian Native American or Alaska Native Multiracial	171 (83.0%) 19 (9.2%) 10 (4.9%) 1 (0.5%) 4 (1.9%)
Ethnicity Hispanic Non-Hispanic	9 (4.4%) 197 (95.6%)
Highest level of education High school diploma, GED, or equivalent Some college Associate degree Bachelor’s degree Graduate degree	29 (14.1%) 44 (21.4%) 24 (11.7%) 84 (40.8%) 25 (12.1%)
Current living situation Own home Rent home/apartment Living with family/friends	106 (51.5%) 70 (34.0%) 29 (14.1%)
Employment status Employed full-time Employed part-time Unemployed Retired Other	144 (69.9%) 36 (17.5%) 10 (4.9%) 10 (4.9%) 3 (1.5%)
Primary mode of transportation Personal vehicle Public transportation Bicycle Walking Other	176 (85.4%) 10 (4.9%) 1 (0.5%) 12 (5.8%) 5 (2.4%)
Do you have health insurance? Yes No	173 (84.0%) 28 (13.6%)
Geographic Setting Rural Urban	38 (18.5) 167 (81.5)
Aware of FTS Yes No	66 (33.3) 132 (66.7)
FTS Usage History Yes No	3 (1.5%) 202 (98.1)
Recreational Drug Use History Yes No	120 (58.3) 85 (41.5)
Age in years, Mean (SD)	46.39 (11.24) Median: 44.5

^a^ Frequencies and percentages may differ due to item non-response.

**Table 2:** Participants’ objective (**a**) and subjective (**b**) knowledge about fentanyl and FTS (*n* = 206).

(a) Objective Knowledge	Mean (SD)
% of Objective Knowledge Questions Answered Correctly	52.73 (25.69)
**Objective Knowledge Items**	***n*** **(%) ^a^**
Fentanyl is an opioid. Correct response: True	156 (75.7)
Fentanyl is 50–100 times stronger than morphine. Correct response: True	150 (72.8)
Fentanyl is only used illegally and has no legitimate medical use. Correct response: False	153 (74.3)
Exposure to even a small amount of fentanyl can be fatal. Correct response: True	161 (78.2)
FTS can detect the presence of fentanyl in other drugs. Correct response: True	133 (64.6)
FTS are an effective harm reduction tool that can help prevent overdoses. Correct response: True	140 (68)
FTS can be used to test substances in which form(s)? Correct Response: All of the above (Powder, Pill, & Liquid)	98 (47.6)
What is the legal status of FTS in the United States? Correct response: FTS are legal to own and use in some states	23 (11.2)
Which substances can FTS detect? Correct response: Fentanyl and most fentanyl analogs	99 (48.1)
How quickly do FTS provide results? Correct response: Within 2 to 5 min	86 (41.7)
How often should you test a drug batch with FTS, even if previous batches were negative? Correct response: Every batch should be tested	113 (54.9)
What can FTS tell you about the amount of fentanyl in a drug? Correct response: They cannot measure the amount	65 (31.1)
FTS costs about $1.00 per test strip. Correct response: True	37 (18.0)
**(b) Subjective Knowledge**	**Mean (SD)**
Self-Rated Knowledge Average Scale Score	2.47 (0.80)
**Subjective Knowledge Items**	***n*** **(%)**
I need more education on fentanyl. Strongly disagree Disagree Neutral Agree Strongly agree	12 (5.9) 15 (7.4) 26 (12.7) 111 (54.4) 40 (19.6)
I am aware of the side effects of using fentanyl. Strongly disagree Disagree Neutral Agree Strongly agree	25 (12.3) 57 (27.9) 30 (14.7) 60 (29.4) 32 (15.7)
I know what a fentanyl overdose looks like. Strongly disagree Disagree Neutral Agree Strongly agree	47 (22.9) 94 (45.9) 21 (10.2) 34 (16.6) 9 (4.4)
I need more knowledge about how to use FTS. Strongly disagree Disagree Neutral Agree Strongly agree	18 (8.8) 22 (10.7) 26 (12.7) 86 (42) 53 (25.9)
I need more training on where to obtain FTS. Strongly disagree Disagree Neutral Agree Strongly agree	21 (10.3) 38 (18.6) 30 (14.7) 75 (36.8) 40 (19.6)
I already have enough knowledge about FTS. Strongly disagree Disagree Neutral Agree Strongly agree	53 (26.4) 93 (46.3) 32 (15.9) 16 (8) 7 (3.5)

^a^ Frequencies and percentages may differ due to item non-response.

**Table 3:** FTS knowledge, perceptions, and intentions across (**a**) usage history and (**b**) sociodemographic subgroups (*n* = 206).

(a) Usage History													
Measures	Mean (SD)												
	Overall		FTS Awareness				FTS Usage			Recreational Drug Use History			
			Yes	No	*p*-Value ^a^		Yes	No	*p*-Value ^a^	Yes		No	*p*-Value ^a^
Objective Knowledge, %	52.73 (25.69)		65.04 (18.71)	47.32 (26.64)	<0.001 *		84.62 (13.32)	52.40 (25.57)	0.025 *	55.26 (23.85)		49.86 (27.47)	0.201
Subjective Knowledge	2.47 (8.0)		2.66 (0.83)	2.38 (0.77)	0.012 *		4.07 (1.00)	2.44 (0.77)	0.012 *	2.49 (0.77)		2.45 (0.84)	0.614
Susceptibility	1.93 (0.57)		2.09 (0.60)	1.84 (0.54)	0.003 *		3.47 (0.52)	1.91 (0.54)	0.004 *	2.07 (0.62)		1.75 (0.43)	<0.001 *
Severity	4.63 (0.38)		4.65 (0.39)	4.64 (0.37)	0.587		4.50 (0.62)	4.63 (0.38)	0.773	4.63 (0.38)		4.63 (0.39)	0.861
Benefits	4.15 (0.84)		4.25 (0.59)	4.15 (0.62)	0.281		4.30 (0.61)	4.17 (0.62)	0.850	4.17 (0.58)		4.19 (0.66)	0.556
Barriers	2.53 (1.14)		2.73 (0.74)	2.64 (0.79)	0.350		2.92 (0.60)	2.67 (0.78)	0.559	2.70 (0.75)		2.66 (0.81)	0.611
Comfort	3.55 (0.87)		3.71 (0.82)	3.48 (0.90)	0.139		3.00 (0.47)	3.56 (0.88)	0.190	3.60 (0.85)		3.50 (0.89)	0.553
Confidence	3.67 (0.69)		3.84 (0.70)	3.60 (0.68)	0.010 *		4.39 (0.73)	3.67 (0.68)	0.097	3.75 (0.62)		3.57 (0.77)	0.182
Intention	2.78 (0.94)		2.84 (0.89)	2.72 (0.98)	0.473		3.28 (0.35)	2.77 (0.94)	0.353	2.90 (0.91)		2.61 (0.97)	0.041 *
**(b) Sociodemographics**													
**Measures**	**Mean (SD)**												
	**Overall**	**Geographic Setting**			**Sex**			**Race**			**Age**		
		**Urban**	**Rural**	***p*** **-Value ^a^**	**Male**	**Female**	***p*** **-Value ^a^**	**White**	**All Other Races ^b^**	***p*** **-Value ^a^**	**≤Median 44.5**	**>Median 44.5**	***p*** **-Value ^a^**
Objective Knowledge, %	52.73 (25.69)	52.97 (25.70)	52.83 (25.62)	0.988	52.11 (26.49)	53.50 (25.03)	0.663	53.44 (25.56)	49.45 (26.47)	0.417	55.12 (26.36)	50.41 (24.91)	0.184
Subjective Knowledge	2.47 (8.0)	2.48 (0.78)	2.42 (0.86)	0.528	2.52 (0.75)	2.41 (0.86)	0.285	2.45 (0.80)	2.58 (0.80)	0.508	2.57 (0.83)	2.37 (0.76)	0.053
Susceptibility	1.93 (0.57)	1.92 (0.55)	1.98 (0.67)	0.839	1.89 (0.56)	2.00 (0.59)	0.149	1.91 (0.58)	2.06 (0.54)	0.034 *	1.99 (0.59)	1.88 (0.56)	0.206
Severity	4.63 (0.38)	4.61 (0.41)	4.74 (0.23)	0.230	4.16 (0.56)	4.62 (0.41)	0.932	4.64 (0.36)	4.60 (0.47)	0.958	4.65 (0.38)	4.62 (0.39)	0.572
Benefits	4.15 (0.84)	4.19 (0.62)	4.13 (0.57)	0.463	4.16 (0.56)	4.17 (0.68)	0.647	4.20 (0.59)	3.99 (0.70)	0.079	4.20 (0.62)	4.14 (0.62)	0.410
Barriers	2.53 (1.14)	2.67 (0.78)	2.80 (0.73)	0.605	2.58 (0.69)	2.80 (0.85)	0.070	2.69 (0.78)	2.63 (0.78)	0.981	2.64 (0.76)	2.71 (0.80)	0.508
Comfort	3.55 (0.87)	3.61 (0.86)	3.29 (0.92)	0.048 *	3.59 (0.82)	3.48 (0.94)	0.424	3.58 (0.87)	3.42 (0.86)	0.286	3.62 (0.79)	3.48 (0.94)	0.368
Confidence	3.67 (0.69)	3.69 (0.70)	3.59 (0.68)	0.193	3.71 (0.69)	3.61 (0.69)	0.327	3.69 (0.67)	3.59 (0.77)	0.526	3.75 (0.73)	3.60 (0.64)	0.048 *
Intention	2.78 (0.94)	2.84 (0.91)	2.56 (1.03)	0.090	2.74 (0.89)	2.81 (1.00)	0.554	2.80 (0.94)	2.68 (0.94)	0.586	2.82 (0.92)	2.74 (0.96)	0.530

Statistical significance at the alpha = 0.05 level indicated by *. ^a^ Mann–Whitney U test. ^b^ Black or African American, Asian, Native American or Alaska Native, Pacific Islander, or Multiracial.

**Table 4:** Perceived barriers to FTS use and access (*n* = 206).

Perceived Barriers	Mean (SD)
Overall Perceived Barriers Score	2.53 (1.14)
**Perceived Barriers Items**	***n*** **(%) ^a^**
Concerns about judgment from others if I buy FTS Strongly disagree Disagree Neutral Agree Strongly agree	49 (24.4) 61 (30.3) 26 (12.9) 56 (27.9) 9 (4.5)
It is too difficult to use FTS Strongly disagree Disagree Neutral Agree Strongly agree	62 (33.2) 77 (41.2) 29 (15.5) 16 (8.6) 3 (1.6)
Using FTS would take too much time Strongly disagree Disagree Neutral Agree Strongly agree	77 (39.9) 80 (41.5) 17 (8.8) 14 (7.3) 5 (2.6)
FTS is too expensive Strongly disagree Disagree Neutral Agree Strongly agree	88 (44.0) 73 (36.5) 10 (5.0) 19 (9.5) 10 (5.0)
Lack of a space where FTS could be conveniently used Strongly disagree Disagree Neutral Agree Strongly agree	74 (38.1) 62 (33.0) 33 (17.0) 20 (10.3) 3 (1.5)
It is difficult to obtain FTS Strongly disagree Disagree Neutral Agree Strongly agree	20 (11.1) 48 (26.7) 54 (30.0) 45 (25.0) 13 (7.2)
FTS is not readily available in stores or pharmacies Strongly disagree Disagree Neutral Agree Strongly agree	15 (8.3) 25 (13.9) 48 (26.7) 67 (37.2) 25 (13.9)
FTS purchased online might not be reliable Strongly disagree Disagree Neutral Agree Strongly agree	15 (7.9) 28 (14.7) 45 (23.7) 73 (38.4) 29 (15.3)
Lack of knowledge about how to use FTS Strongly disagree Disagree Neutral Agree Strongly agree	18 (9.0) 23 (11.5) 30 (15.0) 100 (50.0) 29 (14.5)
Healthcare providers do not support using FTS Strongly disagree Disagree Neutral Agree Strongly agree	36 (20.7) 54 (31.0) 51 (29.3) 26 (14.9) 7 (4.0)
Instructions on how to use FTS are unclear Strongly disagree Disagree Neutral Agree Strongly agree	24 (13.3) 57 (31.7) 54 (30.0) 34 (18.9) 11 (6.1)
Concerns about legality of FTS Strongly disagree Disagree Neutral Agree Strongly agree	32 (16.5) 46 (23.7) 32 (16.5) 60 (30.9) 24 (12.4)
Concerns about being stopped by the police when carrying FTS Strongly disagree Disagree Neutral Agree Strongly agree	36 (18.5) 44 (22.6) 28 (14.4) 60 (30.8) 27 (13.8)

^a^ Frequencies and percentages may differ due to item non-response.

**Table 5:** Predictors of FTS utilization intention (*n* = 206).

Predictors	β	Standardized β	95% CI	*p*-Value
**Model 1 (R^2^ = 0.414, F(df) 19.569 (7), *p* < 0.001) ^a^**				
Objective Knowledge	0.001	0.035	−0.003, 0.006	0.567
Susceptibility	0.456	0.279	0.274, 0.637	<0.001 *
Severity	−0.130	−0.053	−0.414, 0.154	0.369
Benefits	0.269	0.176	0.065, 0.472	0.010 *
Barriers	−0.013	−0.011	−0.163, 0.136	0.861
Comfort	0.467	0.422	0.316, 0.617	<0.001 *
Confidence	0.055	0.039	−0.153, 0.264	0.600
**Model 2 (R^2^ = 0.417, F(df) 11.076 (12), *p* < 0.001) ^b^**				
Objective Knowledge	0.001	0.028	−0.004, 0.006	0.660
Susceptibility	0.442	0.273	0.246, 0.637	<0.001 *
Severity	−0.088	−0.036	−0.381, 0.204	0.551
Benefits	0.250	0.162	0.040, 0.460	0.020 *
Barriers	−0.031	−0.025	−0.187, 0.125	0.695
Comfort	0.453	0.412	0.299, 0.607	<0.001 *
Confidence	0.055	0.039	−0.157, 0.268	0.609

Statistical significance at the alpha = 0.05 level indicated by *. ^a^ Model 1: Multiple linear regression. Dependent variable = intention mean scale score. Predictors = objective knowledge, susceptibility, severity, benefits, barriers, comfort, confidence mean scale scores. ^b^ Model 2: Multiple linear regression. Dependent variable = intention mean scale score. Predictors = objective knowledge, susceptibility, severity, benefits, barriers, comfort, confidence mean scale scores. Controlling for covariates: participant age, race (Black or African American, Asian, Native American or Alaska Native, Pacific Islander, or Multiracial vs. White), sex, geographic setting (rural vs. urban), and recreational drug use history (Yes vs. No). Assumption checks indicated no evidence of autocorrelation, multicollinearity, influential outliers, or heteroskedasticity (Durbin–Watson = 1.94; VIF < 5; Cook’s Distance < 1; modified Breusch–Pagan test *p* = 0.255). The distribution of standardized residuals appeared to support multivariate normality based on the histogram and normal P–P plot, and the scatterplot of standardized residuals versus predicted values suggested that the collective linearity assumption was satisfied.

## Data Availability

The full survey instrument is available in Supplemental File S1. The datasets generated and/or analyzed during the current study are not publicly available due to restrictions within the Institutional Review Board protocol.

## Supplementary Materials

The following supporting information can be downloaded at: https://www.mdpi.com/article/10.3390/healthcare14070833/s1, Supplemental File S1: Survey Instrument; Supplemental File S2: Supplemental Tables.

## Abbreviations

The following abbreviations are used in this manuscript:

CDC	Centers for Disease Control and Prevention
DEA	Drug Enforcement Administration
FTS	Fentanyl test strips
PWUD	People who use drugs
SD	Standard deviation
SAMHSA	Substance Abuse and Mental Health Services Administration
U.S.	United States
