# Supplementary material for: Public Knowledge and Perceptions of Fentanyl Test Strips: A National Cross-Sectional Survey Informed by the Health Belief Model
Source: Healthcare (Basel). 2026 Mar 24;14(7):833. doi: 10.3390/healthcare14070833 (PMC13073353; doi:10.3390/healthcare14070833)
Supplement: Supplementary file 1 [file healthcare-14-00833-s001.zip › Supplemental File S1_survey instrument.pdf]

SUPPLEMENTAL FILE S1: SURVEY INSTRUMENT

Start of Block: Introduction

Public Knowledge and Perceptions Regarding Fentanyl Test Strips (FTS): A National Cross-Sectional Survey

Thank you for your participation in this research study! The purpose of this anonymous survey study is to better understand the general public's knowledge and perceptions regarding fentanyl test strips (FTS) in order to identify knowledge gaps, misconceptions, and barriers to use. Therefore, this survey will ask you about your knowledge and opinions regarding fentanyl test strips (FTS). This will help us develop educational materials aimed at enhancing current knowledge and understanding of fentanyl test strips. This survey consists of 6 sections and should take about 30 minutes to complete. Your participation and responses will be entirely anonymous.

The Information Letter with more details regarding this research study can be reviewed here: INFORMATION LETTER

Clicking the “NEXT” button below serves as your consent to participate in this survey study.

End of Block: Introduction

Start of Block: Demographics

I. INFORMATION ABOUT YOU

What is your gender?

- ○ Male
- ○ Female
- ○ Non-binary
- ○ Other. Please specify: ____
- ○ Prefer not to answer

What is your age in years?

____

What is your race?

- ○ White
- ○ Black or African American
- ○ Asian
- ○ Native American or Alaska Native
- ○ Native Hawaiian or Pacific Islander
- ○ Multiracial. Please specify: ______________________________
- ○ Other. Please specify: ______________________________
- ○ Prefer not to answer

What is your ethnicity?

- ○ Hispanic
- ○ Non-Hispanic
- ○ Prefer not to answer

What is the highest level of school you have completed?

- ○ Less than high school
- ○ High school diploma, GED, or equivalent
- ○ Some college
- ○ Associate degree
- ○ Bachelor's degree
- ○ Graduate degree
- ○ Prefer not to answer

What is your current living situation?

- ○ Own home
- ○ Rent home/apartment
- ○ Living with family/friends
- ○ Unhoused/houseless
- ○ Other. Please specify: ______________________________
- ○ Prefer not to answer

What is your employment status?

- ○ Employed full-time
- ○ Employed part-time
- ○ Unemployed
- ○ Student
- ○ Retired
- ○ Other. Please specify: ____
- ○ Prefer not to answer

What is your primary mode of transportation?

- ○ Personal vehicle
- ○ Public transportation
- ○ Bicycle
- ○ Walking
- ○ Other. Please specify: ____
- ○ Prefer not to answer

In which state do you currently reside?

▼ Alabama ... Prefer not to answer

In which **zip code** do you reside?

____

Do you have health insurance?

- ◯ Yes
- ◯ No
- ◯ Other. Please specify: ____
- ◯ Prefer not to answer

End of Block: Demographics

Start of Block: History of Overdose and Drug Use

Before this survey, had you ever heard of fentanyl test strips (FTS)?

- ◯ Yes
- ◯ No
- ◯ I don't know
- ◯ Prefer not to answer

Have you ever used fentanyl test strips?

- ◯ Yes
- ◯ No
- ◯ I don't know
- ◯ Prefer not to answer

Display This Question:

If Have you ever used fentanyl test strips? = Yes

From where did you obtain fentanyl test strips?

- ◯ Public health department
- ◯ Local pharmacy
- ◯ Doctor's office
- ◯ Hospital or emergency room
- ◯ Drug addiction recovery center
- ◯ Online retailer (e.g., Amazon)
- ◯ Friend or family member
- ◯ Other. Please specify: ____
- ◯ I don't know
- ◯ Prefer not to answer

Have you ever recommended fentanyl tests strips to someone else?

- Yes
- No
- I don't know
- Prefer not to answer

Have you ever used recreational drugs, excluding alcohol, tobacco, or caffeine, for purposes other than those required for medical reasons?

- Yes
- No
- I don't know
- Prefer not to answer

Skip To: End of Block If Have you ever used recreational drugs, excluding alcohol, tobacco, or caffeine, for purposes othe... != Yes

What substances have you used? Please select all that apply.

- ☐ Prescription drugs (non-medical use)
- ☐ Marijuana
- ☐ Cocaine
- ☐ Heroin
- ☐ Methamphetamine
- ☐ Fentanyl
- ☐ Mushrooms
- ☐ LSD (Acid)
- ☐ Hallucinogens
- ☐ MDMA (Ectasy/Molly)
- ☐ Steroids
- ☐ Opioids
- ☐ Ketamine
- ☐ Other. Please specify: ______________________
- ☐ Prefer not to answer

Did you try your first drug before the age of 18?

- ◯ Yes
- ◯ No
- ◯ I don't know
- ◯ Prefer not to answer

Who were you around when you used drugs?

- ◯ Alone
- ◯ Friends
- ◯ Family
- ◯ Strangers
- ◯ Other. Please specify: ____
- ◯ Prefer not to answer

How frequently do you use recreational drugs normally?

- ○ I do not currently use recreational drugs
- ○ A few times a year
- ○ A few times a month
- ○ Once a week
- ○ A few times a week
- ○ Everyday
- ○ Multiple times a day
- ○ Prefer not to answer

Please rate your level of agreement or disagreement with the following statements regarding your history of recreational drug use, using a scale from 1 to 5 where 1= strongly disagree and 5= strongly agree.

	Strongly Disagree	Disagree	Neutral	Agree	Strongly Agree	Not applicable or prefer not to answer
I have tried to quit using drugs, but it was difficult.	○	○	○	○	○	○
I have sought help for drug addiction treatment.	○	○	○	○	○	○
Drugs have negatively affected my relationship with friends and/or family.	○	○	○	○	○	○
I have experienced a near-overdose or a drug overdose in the past.	○	○	○	○	○	○
I have participated in a Syringe Services Program (SSP).	○	○	○	○	○	○
I have used drugs for medical purposes or to self-medicate.	○	○	○	○	○	○

End of Block: History of Overdose and Drug Use

Start of Block: FENTANYL KNOWLEDGE

II. KNOWLEDGE ABOUT FENTANYL AND FENTANYL TEST STRIPS

Please answer the following questions to the best of your ability. If you don't know the answer, that is alright - please choose the option "I don't know."

Fentanyl is an opioid.

- ○ True
- ○ False
- ○ I don't know

Fentanyl is 50 to 100 times more potent than morphine.

- ○ True
- ○ False
- ○ I don't know

Fentanyl is only used illegally (e.g., for recreational purposes) and has no legitimate medical use.

- ○ True
- ○ False
- ○ I don't know

Exposure to even a small amount of fentanyl can be fatal.

- True
- False
- I don't know

Page Break

Definition: Fentanyl is a powerful synthetic opioid that is 50 to 100 times more potent than morphine. Fentanyl, like morphine, is a Schedule II controlled narcotic. It is available as a prescription medication from a doctor for treatment of severe pain (usually pain associated with cancer), but is also available on the street illegally in various forms (pills, powders, injections).

Page Break

Fentanyl test strips (FTS) can detect the presence of fentanyl in other drugs.

- True
- False
- I don't know

Fentanyl test strips are an effective harm reduction tool that can help prevent overdoses.

- True
- False
- I don't know

Fentanyl test strips can be used to test substances in which form(s)? Please select all that apply.

- ☐ Powders
- ☐ Pills
- ☐ Liquid
- ☐ All of the above
- ☐ I don't know

What is the legal status of fentanyl test strips in the United States?

- ○ It is illegal to own and use fentanyl test strips in all states.
- ○ The use of fentanyl test strips is restricted to medical professionals only.
- ○ Fentanyl test strips are legal to own and use in some states.
- ○ Fentanyl test strips are legal to own and use in all states.
- ○ I don't know

What substance(s) can fentanyl test strips detect?

- Fentanyl and most fentanyl analogs
- Only fentanyl
- Only fentanyl analogs
- No substances at all
- I don't know

How quickly do fentanyl test strips provide results?

- Within 2 to 5 minutes.
- Several hours later.
- The next day.
- 2 to 3 business days.
- I don't know

How often should you test a drug batch with fentanyl test strips, even if previous batches were negative?

- Only the first batch needs testing.
- Testing only if the drug looks different.
- Every batch should be tested.
- Testing is unnecessary after one negative batch.
- I don't know

What can fentanyl test strips tell you about the amount of fentanyl in a drug?

- ○ They provide an exact measurement of the amount.
- ○ They give an approximate measurement of the amount.
- ○ They cannot measure the amount.
- ○ I don't know

Fentanyl test strips cost about $1.00 per test strip.

- ○ True
- ○ False
- ○ I don't know

Page Break

Definition: Fentanyl test strips (FTS) are used to detect the presence of fentanyl is various other substances. They can be used by people who use drugs, their family, friends, and caregivers as a harm reduction tool to make sure substances are safe and free from fentanyl. Law enforcement personnel can also use FTS to make sure substances that they come in contact with are safe for them to handle. FTS usually cost about $1.00 per test strip and can be obtained from public health departments, emergency rooms, and online at websites like Amazon or opioid harm reduction websites. Some pharmacies may soon start carrying FTS, but many do not currently. FTS are legal to carry in most states, but not all, so check your particular state before buying them: https://www.networkforphl.org/resources/legality-of-drug-checking-equipment-in-the-united-states/.

End of Block: FENTANYL KNOWLEDGE

Start of Block: Fentanyl Perceived Knowledge

Please rate your level of agreement or disagreement with the following statements about your ***current knowledge*** of fentanyl and fentanyl test strips (FTS), using a scale from 1 to 5 where 1= strongly disagree and 5= strongly agree.

	Strongly Disagree	Disagree	Neutral	Agree	Strongly Agree	I don't know
I need more education on fentanyl. r	○	○	○	○	○	○
I am aware of the side effects of using fentanyl.	○	○	○	○	○	○
I know what a fentanyl overdose looks like.	○	○	○	○	○	○
I need more knowledge about how to use FTS. r	○	○	○	○	○	○
I need more training on where to obtain FTS. r	○	○	○	○	○	○
I already have enough knowledge about FTS.	○	○	○	○	○	○

End of Block: Fentanyl Perceived Knowledge

Start of Block: Perceived Susceptibility to Fentanyl Exposure and Use Risks

III. YOUR BELIEFS ABOUT FENTANYL

Please rate your level of agreement or disagreement with the following statements about **susceptibility to fentanyl use or overdose**, using a scale from 1 to 5 where 1= strongly disagree and 5= strongly agree.

"I believe that…"

	Strongly Disagree	Disagree	Neutral	Agree	Strongly Agree	I don't know or prefer not to answer
Fentanyl overdoses are common in my community	○	○	○	○	○	○
I will likely overdose on fentanyl in my lifetime	○	○	○	○	○	○
I know where to purchase fentanyl	○	○	○	○	○	○
I have friends or family who use fentanyl	○	○	○	○	○	○
I know someone who has overdosed from fentanyl	○	○	○	○	○	○
I have unknowingly or knowingly used fentanyl before	○	○	○	○	○	○
I do not have to worry about overdosing on fentanyl [r]	○	○	○	○	○	○
Even if I use fentanyl, I will not overdose [r]	○	○	○	○	○	○

Please rate your level of agreement or disagreement with the following statements about the **severity of fentanyl use**.

"I believe that…"

	Strongly Disagree	Disagree	Neutral	Agree	Strongly Agree	I don't know or prefer not to answer
Fentanyl can cause a non-fatal overdose	○	○	○	○	○	○
Fentanyl can cause a fatal overdose	○	○	○	○	○	○
Fentanyl can cause serious harm	○	○	○	○	○	○
Using fentanyl can lead to a drug abuse problem	○	○	○	○	○	○
Using fentanyl can cause skin infections	○	○	○	○	○	○
Fentanyl is highly addictive	○	○	○	○	○	○
Using fentanyl can lead to problems holding down a job	○	○	○	○	○	○
Using fentanyl can lead to problems with friends or family	○	○	○	○	○	○

Using fentanyl can lead to legal problems	○	○	○	○	○	○
Using fentanyl can lead to money problems	○	○	○	○	○	○

End of Block: Perceived Susceptibility to Fentanyl Exposure and Use Risks

Start of Block: Perceived Benefits

IV. YOUR BELIEFS ABOUT BENEFITS AND BARRIERS TO USING FENTANYL TEST STRIPS (FTS)

Please rate your level of agreement or disagreement with the following statements about the ***potential benefits*** of fentanyl test strip (FTS) use.

"I believe FTS can…"

	Strongly Disagree	Disagree	Neutral	Agree	Strongly Agree	I don't know or prefer not to answer
Be a useful tool in combating the opioid epidemic	○	○	○	○	○	○
Reduce the risk of overdose	○	○	○	○	○	○
Save lives	○	○	○	○	○	○
Lead to positive changes in a person's drug use	○	○	○	○	○	○
Support recovery from drug addiction	○	○	○	○	○	○
Improve safety for people who use drugs	○	○	○	○	○	○
Improve safety of law enforcement officers	○	○	○	○	○	○
Make a positive impact in my community	○	○	○	○	○	○
Help reduce opioid-related overdoses in my community	○	○	○	○	○	○

End of Block: Perceived Benefits

Start of Block: PERCEIVED BARRIERS TO ACCESSING FTS2

Please rate your level of agreement or disagreement with the following statements about ***barriers*** to accessing and using fentanyl test strips (FTS). ***If certain factors do not apply to you, imagine what might be a barrier for a friend or family member.***

The following factors would make it difficult to use FTS:

	Strongly Disagree	Disagree	Neutral	Agree	Strongly Agree	I don't know or prefer not to answer
Concerns about judgement from others if I buy FTS	○	○	○	○	○	○
It is too difficult to use FTS	○	○	○	○	○	○
Using FTS would take too much time	○	○	○	○	○	○
FTS is too expensive	○	○	○	○	○	○
Lack of a space where FTS could be conveniently used	○	○	○	○	○	○
It is difficult to obtain FTS	○	○	○	○	○	○
FTS is not readily available in stores or pharmacies	○	○	○	○	○	○
FTS purchased online might not be reliable	○	○	○	○	○	○
Lack of knowledge about how to use FTS	○	○	○	○	○	○

Healthcare providers do not support using FTS	○	○	○	○	○	○
Instructions on how to use FTS are unclear	○	○	○	○	○	○
Concerns about legality of FTS	○	○	○	○	○	○
Concerns about being stopped by the police when carrying FTS	○	○	○	○	○	○

What is the **main factor** that could make using FTS easier or clearer?

- ○ Support from healthcare providers
- ○ Clearer instructions
- ○ Availability in more locations (e.g., pharmacies, clinics)
- ○ Legality (e.g., legal to obtain in all states)
- ○ Lower cost or free access
- ○ Increased public awareness and education
- ○ Easier to use test strips (less steps)
- ○ Digital resources (e.g., online tutorials, mobile apps)
- ○ Peer support and testimonials
- ○ Other. Please specify: ____
- ○ Prefer not to answer

End of Block: PERCEIVED BARRIERS TO ACCESSING FTS2

Start of Block: Comfort with FTS (Psychological Safety)

V. COMFORT AND CONFIDENCE ACCESSING AND USING FENTANYL TEST STRIPS (FTS)

Please rate your level of ***comfort*** with the following statements about fentanyl test strip (FTS) use.

	Strongly Disagree	Disagree	Neutral	Agree	Strongly Agree	I don’t know or prefer not to answer
I feel comfortable recommending FTS to friends and/or family	○	○	○	○	○	○
I have a trusted healthcare professional I feel comfortable asking about FTS	○	○	○	○	○	○
I feel comfortable asking my doctor about FTS	○	○	○	○	○	○
I feel comfortable asking my local pharmacist about FTS	○	○	○	○	○	○
I have a trusted individual in my community I feel comfortable asking about FTS	○	○	○	○	○	○
I trust my doctor's ability to provide information on FTS.	○	○	○	○	○	○

I trust my pharmacist's ability to provide information on FTS.	○	○	○	○	○	○
I would not feel embarrassed when asking for FTS.	○	○	○	○	○	○
I trust the FTS results.	○	○	○	○	○	○
I would feel comfortable purchasing FTS in-person in a pharmacy	○	○	○	○	○	○
I would feel comfortable purchasing FTS from an online retailer like Amazon	○	○	○	○	○	○
I would feel comfortable obtaining FTS from my doctor's office	○	○	○	○	○	○
I would feel comfortable obtaining FTS from a hospital emergency room	○	○	○	○	○	○
I would feel comfortable obtaining FTS from a local public health department	○	○	○	○	○	○

I would feel comfortable obtaining FTS from a drug addiction recovery center	o	o	o	o	o	o

End of Block: Comfort with FTS (Psychological Safety)

Start of Block: SELF-EFFICACY IN PERSONAL ACCESS & USE OF FENTANYL-TEST STRIPS (FTS)

Please rate your level of agreement or disagreement with the following statements about your ***confidence in your ability to access or use*** fentanyl test strips (FTS).

	Strongly Disagree	Disagree	Neutral	Agree	Strongly Agree	I don't know or prefer not to answer
I feel confident in my ability to use FTS.	○	○	○	○	○	○
I feel confident in my ability to find more information about FTS.	○	○	○	○	○	○
I know where to purchase FTS.	○	○	○	○	○	○
I feel confident in my ability to find locations where FTS is sold.	○	○	○	○	○	○
If my local pharmacy sold FTS, I would be able to purchase it from them.	○	○	○	○	○	○
I would be able to find online retailers where FTS is sold.	○	○	○	○	○	○

I would be able to use FTS successfully without prior training or reading the box.	○	○	○	○	○	○
I would be able to use FTS after reading the instructions on the box.	○	○	○	○	○	○
I would be able to look up FTS videos on YouTube or social media to help me understand how to use FTS.	○	○	○	○	○	○
I feel confident with my ability to decipher the FTS result.	○	○	○	○	○	○
I know how to proceed after I get results from FTS.	○	○	○	○	○	○

End of Block: SELF-EFFICACY IN PERSONAL ACCESS & USE OF FENTANYL-TEST STRIPS (FTS)

Start of Block: Intention to obtain or use FTS

VI. INTENTION TO USE FENTANYL TEST STRIPS

Please rate your level of agreement or disagreement with the following statements about your ***intention*** to obtain or use fentanyl test strips (FTS).

	Strongly Disagree	Disagree	Neutral	Agree	Strongly Agree	I don't know or prefer not to answer
I intend to obtain FTS in the next 3 months	○	○	○	○	○	○
I would recommend FTS to others who might be at risk	○	○	○	○	○	○
I am likely to use FTS if they are available to me	○	○	○	○	○	○
I am willing to seek out FTS from free community resources	○	○	○	○	○	○
I am willing to pay for FTS if necessary	○	○	○	○	○	○
I am willing to try FTS	○	○	○	○	○	○

End of Block: Intention to obtain or use FTS

Start of Block: Block 11

Thank you so much for your time in completing this survey.

If you or a loved one need help or more information regarding opioid misuse, please call the Substance Abuse and Mental Health Services Administration (SAMHSA) National Helpline at 1-800-662-HELP (4357) or visit their website at https://www.samhsa.gov/find-help/national-helpline.

Please press "NEXT" to submit your survey responses. The survey completion code will be provided on the next screen.

End of Block: Block 11

---

[r] Survey item is reverse coded.
