# Supplementary material for: Public Knowledge and Perceptions of Fentanyl Test Strips: A National Cross-Sectional Survey Informed by the Health Belief Model
Source: Healthcare (Basel). 2026 Mar 24;14(7):833. doi: 10.3390/healthcare14070833 (PMC13073353; doi:10.3390/healthcare14070833)
Supplement: Supplementary file 1 [file healthcare-14-00833-s001.zip › Supplemental File S2_Supplemental Tables.pdf]

SUPPLEMENTAL FILE S2: SUPPLEMENTAL TABLES

Table S1. Perceived Susceptibility to Fentanyl Exposure and Use Risks (N=206)

PERCEIVED SUSCEPTIBILITY	Mean (SD)
Overall Perceived Susceptibility Scale Score	1.93 (0.57)
**Perceived Susceptibility Items**	***n (%)***
**Fentanyl overdoses are common in my community.**	
Strongly disagree	21 (11.2)
Disagree	77 (41.0)
Neutral	37 (19.7)
Agree	41 (12.0)
Strongly agree	12 (6.4)
**I will likely overdose on fentanyl in my lifetime.**	
Strongly disagree	163 (79.1)
Disagree	41 (19.9)
Neutral	1 (0.5)
Agree	1 (0.5)
Strongly agree	0 (0.0)
**I know where to purchase fentanyl.**	
Strongly disagree	146 (71.6)
Disagree	43 (21.2)
Neutral	2 (1.0)
Agree	9 (4.4)
Strongly agree	4 (2.0)
**I have friends or family who use fentanyl.**	
Strongly disagree	128 (65)
Disagree	46 (23.4)
Neutral	11 (5.6)
Agree	10 (5.1)
Strongly agree	2 (1.0)
**I know someone who has overdoses from fentanyl.**	
Strongly disagree	134 (65.7)
Disagree	35 (17.2)
Neutral	3 (1.5)
Agree	15 (17.4)
Strongly agree	17 (8.3)
**I have unknowingly or knowingly used fentanyl before.**	
Strongly disagree	165 (80.5)
Disagree	30 (14.6)
Neutral	4 (2.0)

SUPPLEMENTAL FILE S2: SUPPLEMENTAL TABLES

Agree<br>Strongly agree	5 (2.4)<br>1 (0.5)
**I do not have to worry about overdosing on fentanyl.**<br>Strongly disagree<br>Disagree<br>Neutral<br>Agree<br>Strongly agree	 6 (3.0)<br>17 (8.6)<br>27 (13.7)<br>54 (27.4)<br>93 (47.2)
**Even if I use fentanyl, I will not overdose.**<br>Strongly disagree<br>Disagree<br>Neutral<br>Agree<br>Strongly agree	 71 (38.2)<br>45 (24.2)<br>55 (29.6)<br>10 (5.4)<br>5 (2.7)

Table S2. Perceived Severity of Fentanyl Exposure and Use Risks (N=206)

PERCEIVED SEVERITY	Mean (SD)
Overall Perceived Severity Scale Score	4.63 (0.38)
**Perceived Severity Items**	**n (%)**
**Fentanyl can cause a non-fatal overdose.**<br>Strongly disagree<br>Disagree<br>Neutral<br>Agree<br>Strongly agree	 4 (2.2)<br>11 (5.9)<br>17 (9.1)<br>80 (43.0)<br>74 (39.8)
**Fentanyl can cause a fatal overdose.**<br>Strongly disagree<br>Disagree<br>Neutral<br>Agree<br>Strongly agree	 0 (0.0)<br>0 (0.0)<br>1 (0.5)<br>34 (16.5)<br>166 (82.6)
**Fentanyl can cause serious harm.**<br>Strongly disagree<br>Disagree<br>Neutral<br>Agree<br>Strongly agree	 0 (0.0)<br>0 (0.0)<br>0 (0.0)<br>34 (17.0)<br>166 (83.0)
**Using fentanyl can lead to a drug abuse problem.**	

SUPPLEMENTAL FILE S2: SUPPLEMENTAL TABLES

Strongly disagree<br>Disagree<br>Neutral<br>Agree<br>Strongly agree	0 (0.0)<br>0 (0.0)<br>0 (0.0)<br>42 (21.1)<br>157 (78.9)
**Using fentanyl can cause skin infections.**<br>Strongly disagree<br>Disagree<br>Neutral<br>Agree<br>Strongly agree	 1 (0.9)<br>6 (5.5)<br>43 (39.1)<br>25 (22.7)<br>35 (31.8)
**Fentanyl is highly addictive.**<br>Strongly disagree<br>Disagree<br>Neutral<br>Agree<br>Strongly agree	 0 (0.0)<br>0 (0.0)<br>3 (1.5)<br>59 (30.3)<br>133 (68.2)
**Using fentanyl can lead to problems holding down a job.**<br>Strongly disagree<br>Disagree<br>Neutral<br>Agree<br>Strongly agree	 1 (0.5)<br>0 (0.0)<br>4 (2.0)<br>44 (21.9)<br>152 (75.6)
**Using fentanyl can lead to problems with friends or family.**<br>Strongly disagree<br>Disagree<br>Neutral<br>Agree<br>Strongly agree	 1 (0.5)<br>0 (0.0)<br>3 (1.5)<br>43 (21.3)<br>155 (75.2)
**Using fentanyl can lead to legal problems.**<br>Strongly disagree<br>Disagree<br>Neutral<br>Agree<br>Strongly agree	 1 (0.5)<br>0 (0.0)<br>1 (0.5)<br>48 (24.0)<br>150 (75.0)
**Using fentanyl can lead to money problems.**<br>Strongly disagree<br>Disagree<br>Neutral<br>Agree<br>Strongly agree	 1 (0.5)<br>0 (0.0)<br>2 (1.0)<br>44 (21.9)<br>154 (76.6)

SUPPLEMENTAL FILE S2: SUPPLEMENTAL TABLES

Table S3. Perceived Benefits of FTS (N=206)

PERCEIVED BENEFITS	Mean (SD)
Overall Perceived Benefits Score	4.14 (0.84)
**Perceived Benefits Items**	**n (%)**
**Be a useful tool in combating the opioid epidemic**	
Strongly disagree	1 (0.5)
Disagree	8 (4.0)
Neutral	21 (10.4)
Agree	86 (42.8)
Strongly agree	85 (42.3)
**Reduce the risk of overdose**	
Strongly disagree	1 (0.5)
Disagree	8 (4.0)
Neutral	16 (8.0)
Agree	74 (36.8)
Strongly agree	102 (50.7)
**Save lives**	
Strongly disagree	0 (0.0)
Disagree	4 (2.0)
Neutral	12 (5.9)
Agree	69 (34.0)
Strongly agree	118 (58.1)
**Lead to positive changes in a person's drug use**	
Strongly disagree	5 (2.5)
Disagree	15 (7.6)
Neutral	55 (27.8)
Agree	68 (34.3)
Strongly agree	55 (27.8)
**Support recovery from drug addiction**	
Strongly disagree	6 (3.0)
Disagree	23 (11.7)
Neutral	69 (35.0)
Agree	56 (28.5)
Strongly agree	43 (21.8)
**Improve safety for people who use drugs**	
Strongly disagree	1 (0.5)
Disagree	3 (1.5)
Neutral	18 (8.9)
Agree	65 (32.0)

SUPPLEMENTAL FILE S2: SUPPLEMENTAL TABLES

Strongly agree	116 (57.1)
**Improve safety of law enforcement officers**<br>Strongly disagree<br>Disagree<br>Neutral<br>Agree<br>Strongly agree	 0 (0.0)<br>4 (2.0)<br>14 (7.0)<br>67 (33.7)<br>114 (57.3)
**Make a positive impact in my community**<br>Strongly disagree<br>Disagree<br>Neutral<br>Agree<br>Strongly agree	 0 (0.0)<br>7 (3.6)<br>44 (22.3)<br>84 (42.6)<br>62 (31.5)
**Help reduce opioid-related overdoses in my community**<br>Strongly disagree<br>Disagree<br>Neutral<br>Agree<br>Strongly agree	 0 (0.0)<br>5 (2.5)<br>29 (14.9)<br>77 (39.5)<br>84 (43.1)

Table S4. Comfort Using and Accessing FTS (N=206)

**COMFORT**	**MEAN (SD)**
Overall Comfort Scale Score	3.55 (0.87)
**Comfort Items**	**n (%)**
**I feel comfortable recommending FTS to friends and/or family**<br>Strongly disagree<br>Disagree<br>Neutral<br>Agree<br>Strongly agree	 15 (7.5)<br>36 (7.5)<br>40 (19.9)<br>80 (39.8)<br>30 (14.9)

**I have a trusted healthcare professional I feel comfortable asking about FTS**	
Strongly disagree	20 (9.9)
Disagree	43 (21.2)
Neutral	35 (17.2)
Agree	57 (28.1)
Strongly agree	48 (23.6)
**I feel comfortable asking my doctor about FTS**	
Strongly disagree	21 (10.4)
Disagree	33 (16.4)
Neutral	34 (16.9)
Agree	62 (30.8)
Strongly agree	51 (25.4)
**I feel comfortable asking my local pharmacist about FTS**	
Strongly disagree	22 (10.8)
Disagree	51 (25.0)
Neutral	29 (14.2)
Agree	61 (29.9)
Strongly agree	41 (20.1)
**I have a trusted individual in my community I feel comfortable asking about FTS**	
Strongly disagree	24 (12)
Disagree	53 (26.5)
Neutral	37 (18.5)
Agree	53 (26.5)
Strongly agree	33 (16.5)
**I trust my doctor's ability to provide information on FTS**	
Strongly disagree	4 (2.0)
Disagree	17 (8.5)
Neutral	32 (16.0)
Agree	76 (38.0)
Strongly agree	71 (35.5)

**I trust my pharmacist's ability to provide information on FTS.**	
Strongly disagree	4 (2.0)
Disagree	22 (10.9)
Neutral	40 (19.8)
Agree	74 (36.6)
Strongly agree	62 (30.7)
**I would not feel embarrassed when asking for FTS.**	
Strongly disagree	29 (14.3)
Disagree	55 (27.1)
Neutral	32 (15.8)
Agree	54 (26.6)
Strongly agree	33 (16.3)
**I trust the FTS results.**	
Strongly disagree	3 (1.5)
Disagree	8 (4.1)
Neutral	50 (25.5)
Agree	97 (49.5)
Strongly agree	38 (19.4)
**I would feel comfortable purchasing FTS in-person in a pharmacy**	
Strongly disagree	18 (8.9)
Disagree	40 (19.8)
Neutral	33 (16.3)
Agree	68 (33.7)
Strongly agree	43 (21.3)
**I would feel comfortable purchasing FTS from an online retailer like Amazon**	
Strongly disagree	5 (2.5)
Disagree	25 (12.4)
Neutral	27 (13.4)
Agree	67 (33.2)
Strongly agree	78 (38.6)

**I would feel comfortable obtaining FTS from my doctor's office**<br>Strongly disagree<br>Disagree<br>Neutral<br>Agree<br>Strongly agree	10 (5.0)<br>36 (17.8)<br>27 (13.4)<br>65 (32.2)<br>64 (31.7)
**I would feel comfortable obtaining FTS from a hospital emergency room**<br>Strongly disagree<br>Disagree<br>Neutral<br>Agree<br>Strongly agree	11 (5.4)<br>26 (12.7)<br>27 (13.2)<br>74 (36.3)<br>66 (32.4)
**I would feel comfortable obtaining FTS from a local public health department**<br>Strongly disagree<br>Disagree<br>Neutral<br>Agree<br>Strongly agree	8 (3.9)<br>25 (12.3)<br>32 (15.8)<br>74 (36.5)<br>64 (31.5)
**I would feel comfortable obtaining FTS from a drug addiction recovery center**<br>Strongly disagree<br>Disagree<br>Neutral<br>Agree<br>Strongly agree	15 (7.4)<br>25 (12.3)<br>30 (14.7)<br>72 (35.3)<br>62 (30.4)

Table S5. Confidence in Ability to Use and Access FTS (N=206)

CONFIDENCE	MEAN (SD)

SUPPLEMENTAL FILE S2: SUPPLEMENTAL TABLES

Overall Confidence Scale Score	3.67 (0.69)
**Confidence Items**	**n (%)**
**I feel confident in my ability to use FTS.**	
Strongly disagree	17 (8.5)
Disagree	48 (24.0)
Neutral	34 (17.0)
Agree	73 (36.5)
Strongly agree	28 (14.0)
**I feel confident in my ability to find more information about FTS**	
Strongly disagree	4 (2.0)
Disagree	14 (6.9)
Neutral	15 (7.4)
Agree	71 (34.8)
Strongly agree	100 (49.0)
**I know where to purchase FTS.**	
Strongly disagree	42 (21.2)
Disagree	62 (31.3)
Neutral	39 (19.7)
Agree	41 (20.7)
Strongly agree	14 (7.1)
**I feel confident in my ability to find locations where FTS is sold.**	
Strongly disagree	9 (4.5)
Disagree	21 (10.4)
Neutral	36 (17.8)
Agree	91 (45.0)
Strongly agree	45 (22.3)

**If my local pharmacy sold FTS, I would be able to purchase it from them.**	
Strongly disagree	4 (2.0)
Disagree	5 (2.6)
Neutral	26 (13.3)
Agree	87 (44.4)
Strongly agree	74 (37.8)
**I would be able to find online retailers where FTS is sold.**	
Strongly disagree	2 (1.0)
Disagree	5 (2.5)
Neutral	25 (12.6)
Agree	77 (38.7)
Strongly agree	90 (45.2)
**I would be able to use FTS successfully without prior training or reading the box.**	
Strongly disagree	51 (25.9)
Disagree	66 (33.5)
Neutral	44 (22.3)
Agree	21 (10.7)
Strongly agree	15 (7.6)
**I would be able to look up FTS videos on YouTube or social media to help me understand how to use FTS.**	
Strongly disagree	3 (1.5)
Disagree	2 (1.0)
Neutral	16 (7.8)
Agree	84 (41.2)
Strongly agree	99 (48.5)
**I feel confident with my ability to decipher the FTS result.**	
Strongly disagree	7 (3.5)
Disagree	17 (8.5)
Neutral	29 (14.6)
Agree	86 (43.2)
Strongly agree	60 (30.2)

**I know how to proceed after I get results from FTS.**	
Strongly disagree	13 (6.8)
Disagree	29 (15.1)
Neutral	48 (25.0)
Agree	66 (34.4)
Strongly agree	36 (18.8)

Table S6. FTS Utilization Intentions (N=206)

INTENTION	MEAN (SD)
Intention to Use FTS Average Scale Score	2.78 (0.94)
**INTENTION ITEMS**	***n* (%)**
**I intend to obtain FTS in the next 3 months.**	
Strongly disagree	116 (57.4)
Disagree	63 (31.2)
Neutral	15 (7.4)
Agree	7 (3.5)
Strongly agree	1 (0.5)
**I would recommend FTS to others who might be at risk.**	
Strongly disagree	21 (10.3)
Disagree	9 (4.4)
Neutral	33 (16.2)
Agree	72 (35.3)
Strongly agree	69 (33.88)
**I am likely to use FTS if they are available to me.**	
Strongly disagree	84 (41.4)
Disagree	46 (22.7)
Neutral	55 (27.1)
Agree	15 (7.4)
Strongly agree	3 (1.5)
**I am willing to seek out FTS from free community resources.**	
Strongly disagree	47 (23.4)
Disagree	29 (14.4)
Neutral	51 (25.4)
Agree	54 (26.9)
Strongly agree	20 (10)

SUPPLEMENTAL FILE S2: SUPPLEMENTAL TABLES

**I am willing to pay for FTS if necessary.**	
Strongly disagree	36 (17.6)
Disagree	21 (10.2)
Neutral	40 (19.5)
Agree	78 (38)
Strongly agree	30 (14.6)
**I am willing to try FTS.**	
Strongly disagree	33 (16.2)
Disagree	23 (11.3)
Neutral	52 (25.5)
Agree	70 (34.3)
Strongly agree	26 (12.7)
